# Role of the methionine cycle in the temperature‐sensitive responses of potato plants to potato virus Y

**DOI:** 10.1111/mpp.13009

**Published:** 2020-11-04

**Authors:** Igor Fesenko, Nadezhda Spechenkova, Anna Mamaeva, Antonida V. Makhotenko, Andrew J. Love, Natalia O. Kalinina, Michael Taliansky

**Affiliations:** ^1^ Shemyakin‐Ovchinnikov Institute of Bioorganic Chemistry of the Russian Academy of Sciences Moscow Russian Federation; ^2^ Belozersky Institute of Physico‐chemical Biology Lomonosov Moscow State University Moscow Russian Federation; ^3^ The James Hutton Institute Invergowrie, Dundee UK

**Keywords:** isobaric tags for relative and absolute quantitation (iTRAQ), methionine cycle, potato virus Y, proteomic analysis, temperature‐dependent antivirus defence, virus susceptibility

## Abstract

Plant–virus interactions are greatly influenced by environmental factors such as temperatures. In virus‐infected plants, enhanced temperature is frequently associated with more severe symptoms and higher virus content. However, the mechanisms involved in such regulatory effects remain largely uncharacterized. To provide more insight into the mechanisms whereby temperature regulates plant–virus interactions, we analysed changes in the proteome of potato cv. Chicago plants infected with potato virus Y (PVY) at normal (22 °C) and elevated temperature (28 °C), which is known to significantly increase plant susceptibility to the virus. One of the most intriguing findings is that the main enzymes of the methionine cycle (MTC) were down‐regulated at the higher but not at normal temperatures. With good agreement, we found that higher temperature conditions triggered consistent and concerted changes in the level of MTC metabolites, suggesting that the enhanced susceptibility of potato plants to PVY at 28 °C may at least be partially orchestrated by the down‐regulation of MTC enzymes and concomitant cycle perturbation. In line with this, foliar treatment of these plants with methionine restored accumulation of MTC metabolites and subverted the susceptibility to PVY at elevated temperature. These data are discussed in the context of the major function of the MTC in transmethylation processes.

## Graphical abstract

The work describes mechanisms whereby proteomic, transcriptional, and metabolic changes associated with the methionine cycle may modulate temperature‐sensitive plant–virus interactions.

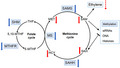

## 1 INTRODUCTION

Potato (*Solanum tuberosum*) plants, like all other crop plants, are constantly exposed to various pathogenic agents such as bacteria, fungi, oomycetes, nematodes, and viruses. This results in severe crop losses, which ultimately leads to a decline in food production worldwide. Among the various plant pathogens, viruses account for up to 50% of all novel/emerging plant diseases (Whitfield et al., 2015). Potato virus Y (PVY) is one of the most common pathogens of Solanaceae family members, including potato. The most effective and reliable method of plant protection is by increasing plant resistance to viruses.

Although plants have evolved multilayered surveillance and defence mechanisms to resist virus infections, it is worth noting that elevated temperatures may suppress a range of these antiviral responses, rendering plants more susceptible to virus attack. Such a phenomenon has been observed in incompatible plant–virus interactions such as *R* (resistance) gene‐mediated defence responses (Zhu et al., 2010). For example, the resistance mediated in tobacco by the *N* gene (a resistance [*R*] gene encoding a nucleotide‐binding site leucine‐rich repeat [NBS‐LRR] domain‐containing protein) against tobacco mosaic virus (TMV) confers defence only at temperatures below 28 °C. Under these conditions, TMV triggers a hypersensitive response (HR), which is defined as a rapid necrosis at the site of virus entry that prevents further spread of the pathogen to cells surrounding the initial site of infection. At higher temperatures, resistance does not develop and TMV spreads systemically throughout the plant (Zhu et al., 2010). The observed deregulation of defence responses in a temperature‐dependent manner occurs presumably due to temperature‐sensitive conformational loss of function of the tobacco NBS‐LRR protein, preventing its interaction with the TMV p50 effector protein (Zhu et al., 2010).

A variety of *R* genes conferring HR to PVY have been identified in potato species (*Solanum* spp.) (Solomon‐Blackburn and Bradshaw, 2007). Many of these genes, including *Ny* in *S*. *sparsipilum* and *S. sucrense*, or *Ny‐1* in potato cv. Rywal, confer resistance only at low temperatures (16–20 °C). At higher temperatures (24–28 °C), resistance does not take place, and PVY infects plants systemically. In contrast, resistance genes *Ry_sto_* in *S. stoloniferum* and *Ry_chc_* in *S. chacoense*, which confer extreme resistance (inhibit virus replication without apparent HR) to the tobacco veinal necrosis strain of PVY (PVY^N^), are functional at both low (16–20 °C) and elevated (above 24 °C) temperatures (Bradshaw and Ramsay, 2005; Solomon‐Blackburn and Bradshaw, 2007).

Compatible virus infections, which are characterized by efficient systemic virus spread from the initially infected tissues, are also affected by higher temperatures (Anfoka et al., 2016; Obrępalska‐Stęplowska et al., 2015; Prasch and Sonnewald, 2013). For instance, elevated temperatures significantly increase the susceptibility of *Arabidopsis* plants to turnip mosaic virus (Prasch and Sonnewald, 2013). Likewise, tomato plants subjected to higher temperatures were more susceptible to tomato yellow leaf curl virus (Anfoka et al., 2016). With regard to PVY, we have recently shown that susceptibility of potato cv. Chicago plants to systemic PVY infection (virus accumulation and symptom production in systemically infected leaves) is significantly enhanced by elevated temperature compared with normal conditions (Makarova et al., 2018). Interestingly, such an enhanced susceptibility was correlated with reduced expression of genes encoding pathogenesis‐related (PR) proteins, which are hallmarks of salicylic acid (SA)‐mediated plant defence responses. We have also demonstrated that SA pretreatment subverts enhanced susceptibility to PVY in cv. Chicago at higher temperature (Makarova et al., 2018), implicating SA as a key regulator of mechanisms determining susceptibility/resistance in potato (Baebler et al., 2011; Carr et al., 2018; Kogovšek and Ravnikar, 2013; Love et al., 2005; Vlot et al., 2009).

RNA interference (RNAi), which is also called RNA silencing, is a versatile, evolutionarily conserved and sequence‐specific mechanism for controlling endogenous gene expression and degrading foreign nucleic acids. RNAi‐based defence responses involve processing of virus‐derived double‐stranded RNAs (dsRNAs) into small interfering RNAs (siRNAs), which in a complex with some plant proteins trigger the sequence‐specific inactivation/degradation of viral RNAs (Baulcombe, 2005; Ding, 2010; Guo et al., 2019; Mlotshwa et al., 2008; Yang and Li, 2018). Remarkably, these RNAi‐mediated mechanisms may also be controlled by temperature. Sometimes RNAi‐based antivirus defences are enhanced by elevated temperatures, which may therefore increase the resistance to virus infection (Chellappan et al., 2005; Szittya et al., 2003; Tuttle et al., 2008). However, this situation is complicated because both RNAi (Lewsey et al., 2010; Zhou et al., 2014) and the activity of virus‐encoded silencing (RNAi) suppressors can be tightly interrelated with SA‐dependent signalling pathways (Csorba et al., 2015; Laird et al., 2013; Love et al., 2012), which may also consequently be controlled by temperature (Shams‐Bakhsh et al., 2007).

Interestingly, antiviral defence mechanisms may also be regulated via the interplay of viruses with the plant methylation cycle (MTC; Mäkinen and De, 2019). In the MTC, S‐adenosyl methionine synthase (SAMS) converts methionine (MET) and adenosine triphosphate to S‐adenosyl methionine (SAM), which is a universal methyl donor for numerous methylation reactions. As a result of transferring its methyl groups to target molecules, SAM becomes S‐adenosyl homocysteine (SAH), which is then converted to homocysteine (HCY) by SAH hydrolase (SAHH). MET synthase (MS) completes the cycle by converting HCY back to MET with 5‐methyltetrahydrofolate (5‐MTHF) as methyl group donor (reviewed by Mäkinen and De, 2019). 5‐MTHF is a product of the MTC‐coupled folate cycle synthesized from 5,10‐MTHF by methylenetetrahydrofolate reductase (MTHFR). In turn, 5,10‐MTHF is converted from tetrahydrofolate by serine hydroxymethyltransferase (SHM).

The MTC plays a central role in various biological processes such as metabolism, signal transduction, and gene expression. With regard to virus–plant interactions, the MTC is closely linked to RNAi pathways, in which siRNAs are stabilized by the MTC‐based methylation process (Li et al., 2005). Plant host DNA methylation, as an epigenetic mechanism driven by the MTC, has also been suggested as playing an important role in modulating host responses to viruses by modifying functions of host genes and affecting gene expression (Corrêa et al., 2020; Wang et al., 2019). Another factor released as a product of the MTC‐related pathway is ethylene, a plant hormone that, like other hormones, plays important roles in plant responses to biotic and abiotic stress responses (reviewed in Müller and Munné‐Bosch, 2015).

To provide more insight into the mechanisms of how temperature regulates plant antiviral defence responses, this study used an isobaric tag for relative and absolute quantitation (iTRAQ) labelling to comprehensively analyse changes in the proteome of potato plants (cv. Chicago) infected with PVY at normal (22 °C) and elevated temperature (28 °C) conditions. We showed that at 14 days postinoculation (dpi) infection at 28 °C (which significantly facilitates susceptibility to the virus) induced greater changes in the abundance of proteins (152 differentially expressed proteins, DEPs) than at 22 °C (23 DEPs). Gene ontology (GO) and Kyoto Encyclopedia of Genes and Genomes (KEGG) pathway analyses were applied to characterize the protein functions and the significant pathways associated with the DEPs, and protein–protein interaction networks were constructed using STRING. It was not surprising that biotic and abiotic stress responsive proteins were among the groups up‐regulated during PVY infection at both normal and higher temperature.

Most strikingly, the main MTC enzymes such as MS, SAMS, and SAHH, as well as enzymes of the MTC‐coupled folate cycle, namely SHM and MTHFR, were found to be down‐regulated in potato plants infected with PVY (at 8 dpi), but only at the higher and not at normal temperatures. In good agreement, we found that these conditions (PVY + higher temperature) triggered consistent and concerted changes in levels of consecutive MTC precursors of SAM, including HCY and MET, and the downstream enzyme SAHH. Thus, we hypothesize that the enhanced susceptibility of potato plants to PVY observed at 28 °C may be orchestrated at least partially by down‐regulation of MTC enzymes disturbing normal functioning of the cycle. In line with this, foliar treatment of these plants with MET restored accumulation of MTC metabolites and subverted the susceptibility to PVY at elevated temperature in the potato plants, supporting the importance of MTC as a key component of antiviral defence.

## 2 RESULTS

### 2.1 Impact of elevated temperature on the susceptibility of potato plants to PVY

In previous work, we studied the effect of moderately elevated temperature (28 °C) on the dynamics of PVY infection in potato plants (Makarova et al., 2018). This temperature was chosen to mimic the conditions that may arise during mild heat stress under global climate changes. In inoculated leaves of cv. Chicago, PVY was detected by 3 dpi in inoculated leaves at relatively low levels, which did not increase significantly over time and were not affected by temperature (Figure 2b in Makarova et al., 2018). Starting at approximately 5–8 dpi, PVY spread systemically, invading upper leaves at both normal (22 °C) and elevated (28 °C) temperatures. With time, a significant increase in virus levels was observed in the systemically infected leaves of plants grown at 22 °C (up to seven‐fold); however, virus levels were found to be significantly higher in corresponding tissues of plants grown at 28 °C (Makarova et al., 2018), suggesting that elevated temperature greatly enhances susceptibility of cv. Chicago plants to PVY.

**FIGURE 1:**
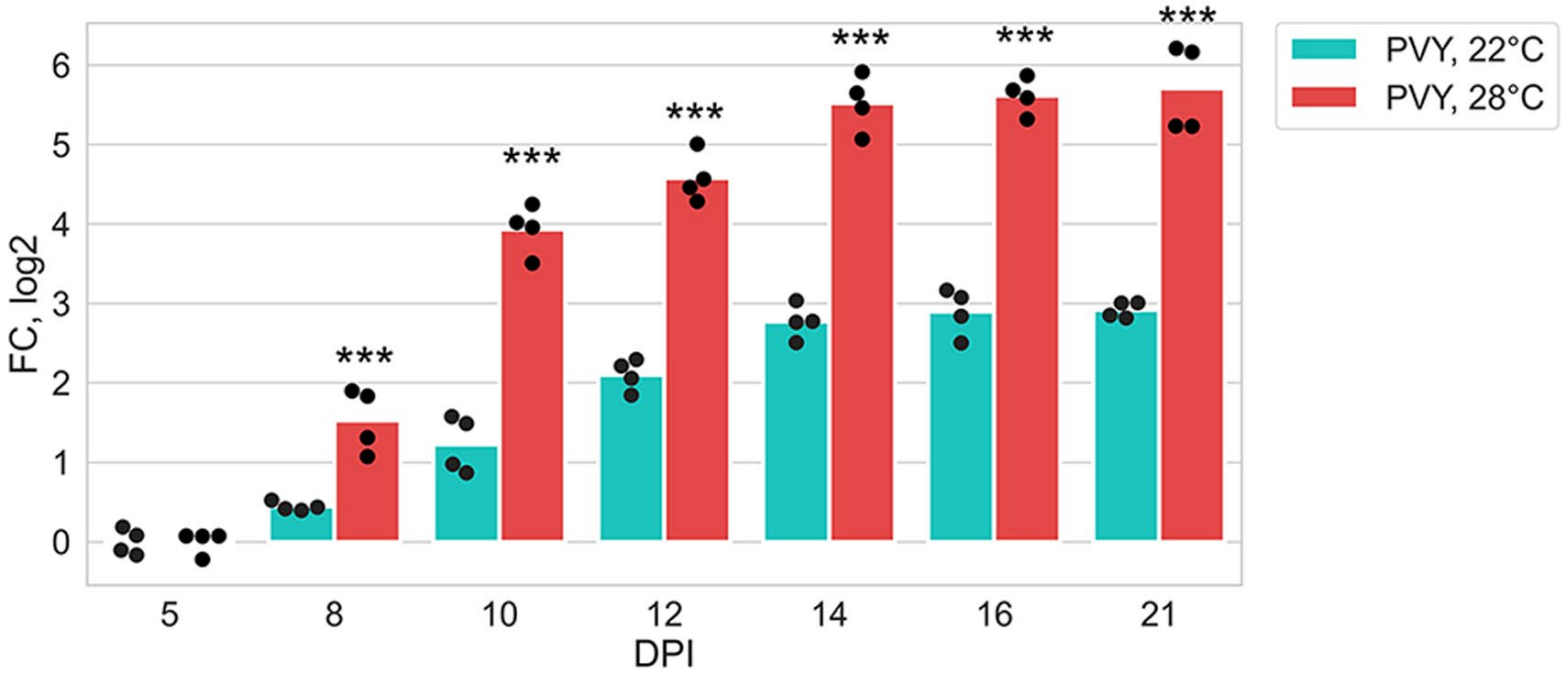
Accumulation of PVY RNA (measured using quantitative reverse transcription PCR) in systemically infected leaves of potato plants over 3–21 days postinoculation (dpi) time periods at 22 or 28 °C as shown. Analysis of variance and Tukey's HSD post hoc tests were performed for quantitative reverse transcription PCR data. ***p < .001

More detailed time course experiments carried out in this work (Figure 1) confirmed this suggestion and revealed two time points that may represent critical stages in determining the temperature sensitivity of potato plant susceptibility to PVY: an early time point (8 dpi) at which PVY accumulation undergoes a temperature‐dependent divergence and a later time point that represents maximal accumulation of the virus (14 dpi) (Figure 1). These time points were selected for further proteomic analysis.

### 2.2 Protein profiles of PVY‐infected potato plants at normal and elevated temperature

**FIGURE 2:**
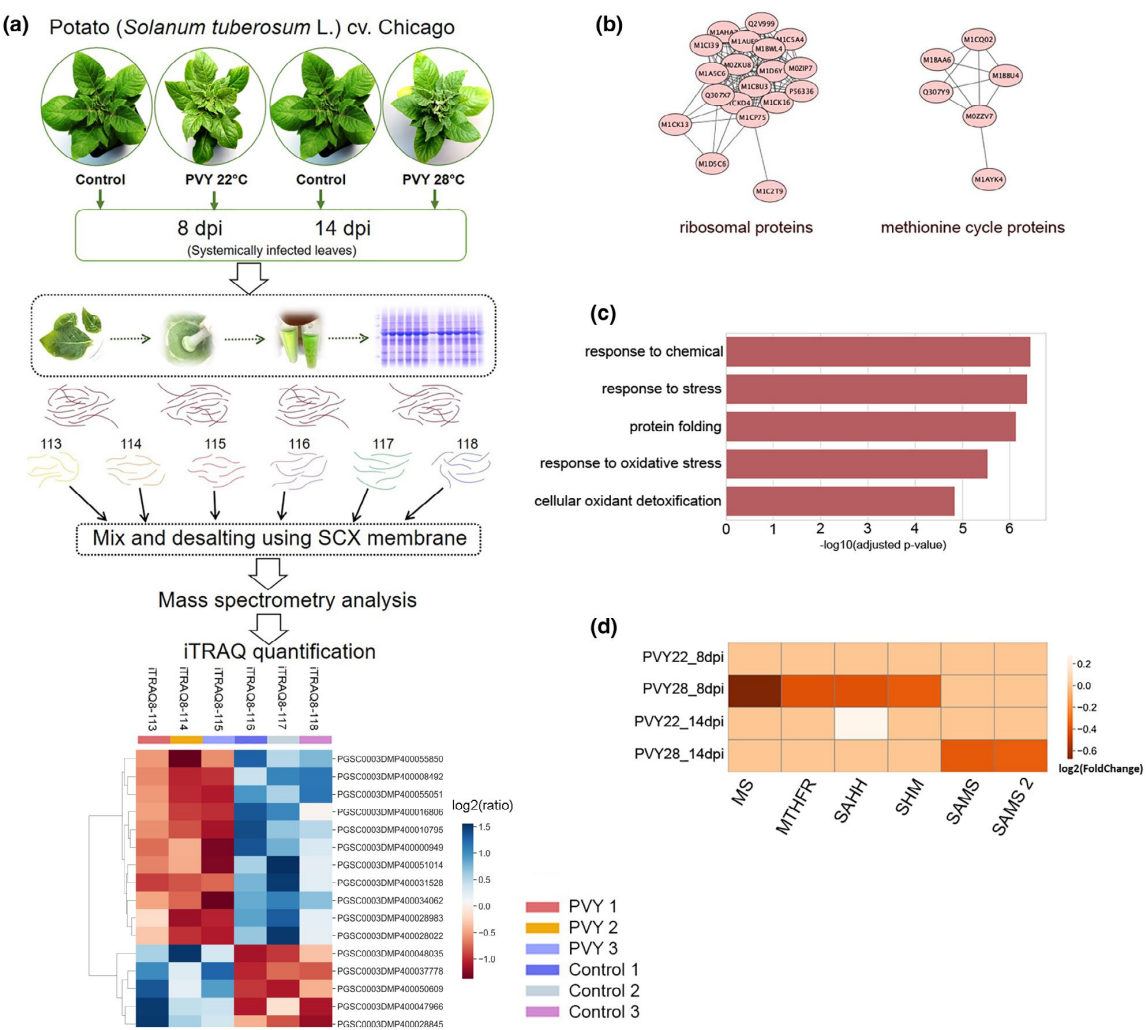
(a) The strategy for comparative quantitative analysis of protein expression in potato leaves by 8‐plexisobaric tags for relative and absolute quantitation (iTRAQ). For detailed iTRAQ data see Tables S2 and S3. (b) Protein–protein interaction networks of differentially expressed proteins (DEPs). Proteins are indicated with nodes, and interactions between proteins are represented by edges. M1CQ02‐MS; M1BBU4‐MTHFR; M0ZZV7–SAHH; Q307Y9‐SAMS 1; M1BAA6‐SHM; M1AYK4‐S‐formylglutathione hydrolase. (c) GO enrichment analysis of the up‐regulated DEPs in infected potato plants at 14 days postinoculation (dpi) at 28 °C. (d) Heatmap showing the changes in abundance of key enzymes of the methionine cycle (MTC) and MTC‐related folate cycle. PVY22_8dpi, infected potato plants at 8 dpi at 22 °C; PVY28_8dpi, infected potato plants at 8 dpi at 28 °C; PVY22_14dpi, infected potato plants at 14 dpi at 22 °C; PVY28_8dpi, infected potato plants at 14 dpi at 28 °C

To explore the underlying mechanisms that lead to increased susceptibility of potato to PVY at elevated temperature compared with normal conditions, iTRAQ‐based quantitative comparative proteomic analysis was conducted at 8 and 14 dpi (Figure 2a).

A total of 22,615 peptides were identified with a 1% false discovery rate (FDR) (Table S2), which were assigned to 5,756 proteins from a custom database (Methods S1). The numbers of peptides and corresponding protein groups identified by liquid chromatography tandem mass spectrometry (LC–MS/MS) analysis for both control mock‐inoculated and plants infected with PVY at 22 or 28 °C are indicated in Table 1. Differential protein screening (determined by the ratio in the PVY‐infected samples and corresponding untreated control (mock‐inoculated plants at 22 °C) identified 642 DEPs.

**TABLE 1:** Numbers of peptides and corresponding protein groups identified by liquid chromatography tandem mass spectrometry (LC–MS/MS)

	PVY 22 °C 8 dpi	PVY 28 °C 8 dpi	PVY 22 °C 14 dpi	PVY 28 °C 14 dpi
Peptides	14,362	12,923	12,409	12,876
Protein groups	2,625	2,333	2,539	2,505

Abbreviation: dpi, days postinoculation.

In PVY‐infected plants grown at normal temperature, we identified only 16 DEP groups including five up‐regulated and 11 down‐regulated proteins at 8 dpi and 23 DEP protgroups (18 up‐regulated and five down‐regulated) at 14 dpi (Table S3). This suggests that at this temperature the response of the plants to PVY was relatively moderate. At 8 dpi, among the population of up‐regulated DEPs, we found biotic and abiotic stress‐responsive proteins such as superoxide dismutase (SOD), which plays a vital role in protecting plants from reactive oxygen species (ROS)‐mediated injury (Younus, 2018), and fibrillin 8, a plastid‐associated lipid‐binding protein induced by abscisic acid (ABA) (Yang et al., 2006), whereas the down‐regulated DEPs included ribosomal proteins, glutamine cyclotransferase, and monodehydroascorbate reductase (Table S3). Both glutamine cyclotransferase and monodehydroascorbate reductase have been shown to mitigate damage in plants under abiotic stress (Johnston et al., 2015; Paulose et al., 2013).

At 14 dpi in virus‐infected plants, we identified 18 up‐regulated DEPs, which included a group of ribosomal proteins and also α‐tubulin, the latter of which constitutes part of the cytoskeleton (Table S3). This is consistent with previous findings that have implicated cytoskeletal components in intra‐ and intercellular trafficking of many viruses (Pitzalis and Heinlein, 2017). In addition, we also observed that SOD was down‐regulated in infected plants, which presumably means reduced protection against ROS damage (Younus, 2018) at this stage of infection.

At elevated temperature, the proteome changes were more pronounced. In virus‐infected plants at 8 dpi, we identified 64 DEP groups, 15 of which were up‐regulated and 49 down‐regulated. Among the up‐regulated protein groups, we identified stress response‐related proteins such as calreticulin, salt tolerance protein 4, acidic endochitinase, and patatin 3 (Table S3 **).** Given the abundance of the down‐regulated proteins more in‐depth characterization was performed. The g:Profiler (http://biit.cs.ut.ee/gprofiler/) webtool was used for finding enriched GO terms in differentially regulated protein groups. According to GO enrichment analysis, down‐regulated proteins were mainly assigned to such biological processes as translation (GO:0,006,412), cellular amide metabolism (GO:0,043,603), and amide biosynthesis (GO:0,043,604). According to KEGG pathway analysis, the down‐regulated proteins were enriched in ribosome (KEGG:03,010) and metabolic pathways (KEGG:01100) (Table S4). We then used the STRING database to create functional protein association networks of DEPs down‐regulated at elevated temperature. Based on this analysis, two distinct clusters were identified: ribosomal proteins and proteins involved in the MTC (Figure 2b). In the MTC cluster, the main enzymes of the MTC were identified: MS, SAMS, and SAHH. Moreover, this cluster included some MTC‐associated proteins such as those involved in the folate cycle: SHM and MTHFR. As mentioned above, aspects of the MTC play an important role in plant defence against viral infection, and down‐regulation of these enzymes presumably leads to disturbances in proper functioning of the MTC, which in turn may cause enhanced susceptibility to PVY at higher temperature. With regard to the down‐regulation of ribosomal proteins, this suggests that PVY infection under elevated temperature could inhibit translational processes.

PVY‐infected potato plants at 14 dpi at the higher temperature had 79 up‐regulated protein groups. The most up‐regulated proteins belonged to defence proteins and included β‐glucanases, pathogen‐ and wound‐inducible antifungal proteins, acidic endochitinase, and a class II chitinase. Chitinases play an important role in defence against fungi and insects, but proteomic studies have revealed they are also differentially regulated during viral infection. The GO term analysis showed enrichment in the following biological processes: responses to stress (GO:0,006,950), protein folding (GO:0,006,457), and oxidative stress responses (GO:0,006,979) (Table S4 and Figure 2c). According to KEGG pathways analysis, protein processing in the endoplasmic reticulum (KEGG:04,141) and glutathione metabolism (KEGG:00,480) were enhanced (Table S4). We also identified 73 down‐regulated DEPs. Among the top 10 down‐regulated DEPs, chloroplast proteins and histone H2A were observed. According to GO term analysis, down‐regulated proteins were mainly enriched in such biological processes as photosynthesis (GO:0,015,979), tetrapyrrole biosynthetic processes (GO:0,033,014), photosynthesis, and light harvesting (GO:0,009,765). The KEGG pathways of down‐regulated DEPs were enriched in porphyrin and chlorophyll metabolism (KEGG:00,860), and biosynthesis of secondary metabolites (KEGG:01110).

Interestingly, some commonly identified proteins showed a different pattern of changes across 8 and 14 dpi. The abundance of acidic endochitinase (PGSC0003DMP400056271) and patatin 3 (PGSC0003DMP400017707), which are involved in response to pathogens in potato, increased at 14 dpi in comparison with 8 dpi. The chaperone DnaJ (PGSC0003DMP400040462) was down‐regulated at 8 dpi but up‐regulated at 14 dpi.

One of the most striking findings of the proteomic analysis is therefore that all key enzymes of the MTC and MTC‐related folate cycle, including MS, SAMS, SAHH, SHM, and MTHFR, were down‐regulated at the protein level by PVY infection at the higher temperature (Figure 2d).

### 2.3 RNA expression levels of key MTC‐related genes

To examine whether the MTC‐related proteomic changes identified above were due to transcriptional regulation, we examined how PVY infection at the higher temperature might alter gene expression of *MS*, *SAMS*, and *SAHH*, all key components of the plant‐activated MTC, and *SHM* and *MTHFR*, which are associated with the MTS‐coupled folate cycle.

**FIGURE 3:**
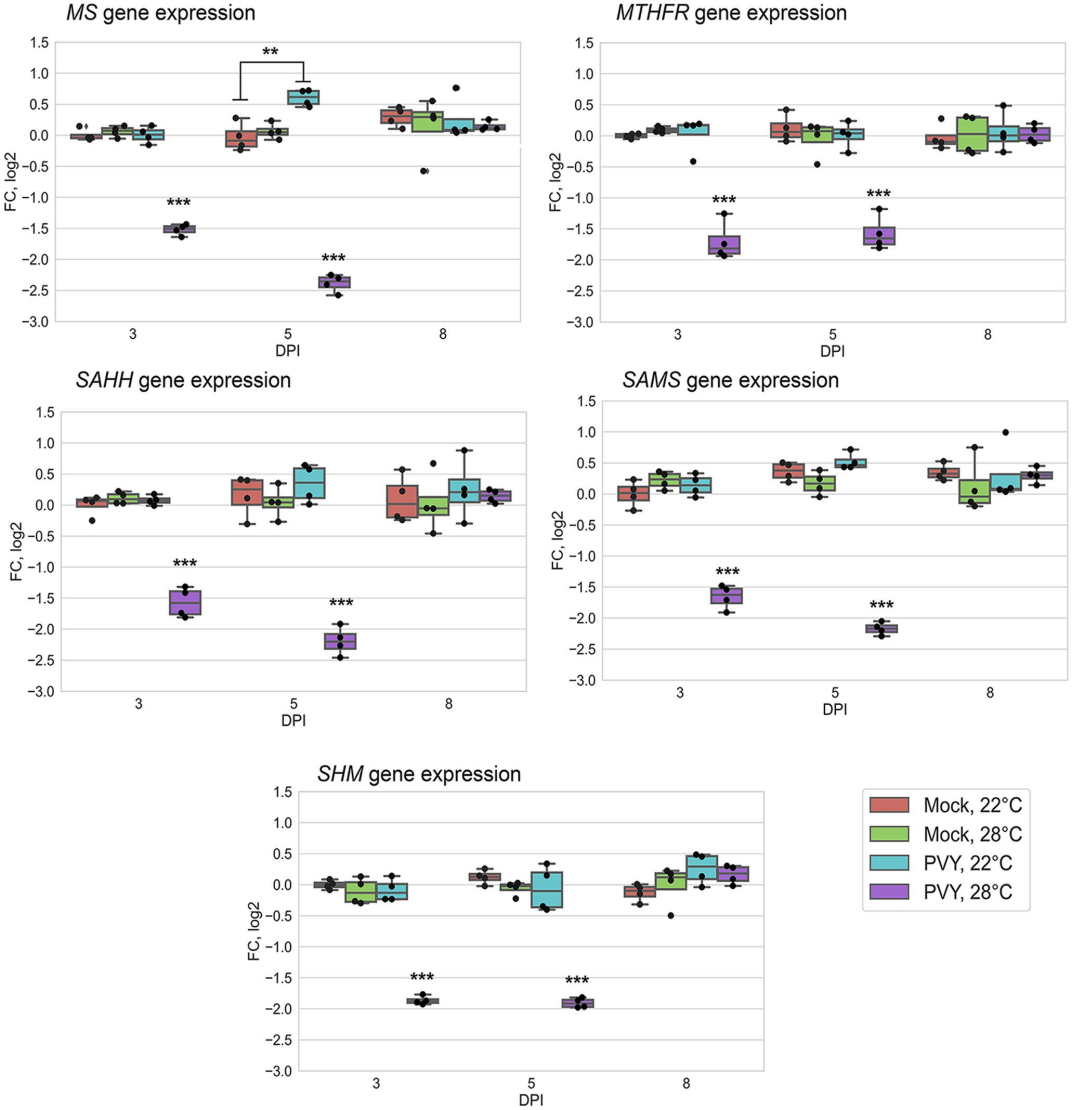
Expression level of methionine cycle (MTC) and MTC‐related protein genes: *StMS* (a) and *StSAMS* (b), *StSAHH* (c), *StSHM* (d) and *StMTHFR* (e) (measured using quantitative reverse transcription PCR [RT‐qPCR]) in systemically infected leaves of potato plants at 22 or 28 °C at 5, 7, and 8 days postinoculation (dpi) as shown. Analysis of variance and Tukey's HSD post hoc tests were performed for RT‐qPCR data. **p < .01, ***p < .001

Quantitative reverse transcription PCR (RT‐qPCR) analysis clearly showed that systemic PVY infection at elevated temperature (28 °C) significantly, but temporarily, reduced *MS*, *SAMS*, *SAHH, SHM*, and *MTHFR* transcription at 5–7 dpi (Figure 3). Interestingly, these transcriptional changes just preceded the sharp growth of PVY accumulation at 28 °C compared with 22 °C (Figure 3), which presumably triggered the coincident decrease in accumulation of MS, SAMS, SAHH, SHM, and MTHFR enzymes at a slightly later stage (8 dpi).

In contrast, at normal temperature (22 °C) PVY did not affect transcription of *SAMS*, *SAHH, SHM*, and *MTHFR*, but moderately enhanced *MS* transcript accumulation (at 7 dpi). Thus, under these conditions, there is a disparity between *MS* gene transcription and translation levels (compare Table S3 and Figure 3). In fact, inconsistency between proteomic and transcriptomic data have been previously well documented (Li et al., 2015; Zhu et al., 2018), which may in part be accounted for by the fact that aside from gene transcription, additional factors such as posttranscriptional regulation (including translational and posttranslational control), selective degradation of mRNAs, and protein turnover can greatly influence the level of final protein accumulation.

Interestingly, the higher temperature (28 °C) did not affect expression of the genes encoding the MTC‐related enzymes in uninfected plants compared with 22 °C, suggesting that alterations in the RNA transcript levels of these genes are a consequence of the integrated response of potato plants to both PVY infection and elevated temperature.

Collectively, these data imply that concomitant changes in the expression of the major enzymes of the MTC and MTC‐related folate cycles, including MS, SAMS, SAHH, SHM, and MTHFR, all of which are down‐regulated by PVY at 28 °C, may reduce accumulation levels of the key MTC metabolites, which could perturb a wide range of transmethylation processes. This in turn may influence susceptibility of potato plants to PVY under these conditions.

### 2.4 Accumulation of MTC metabolites

**FIGURE 4:**
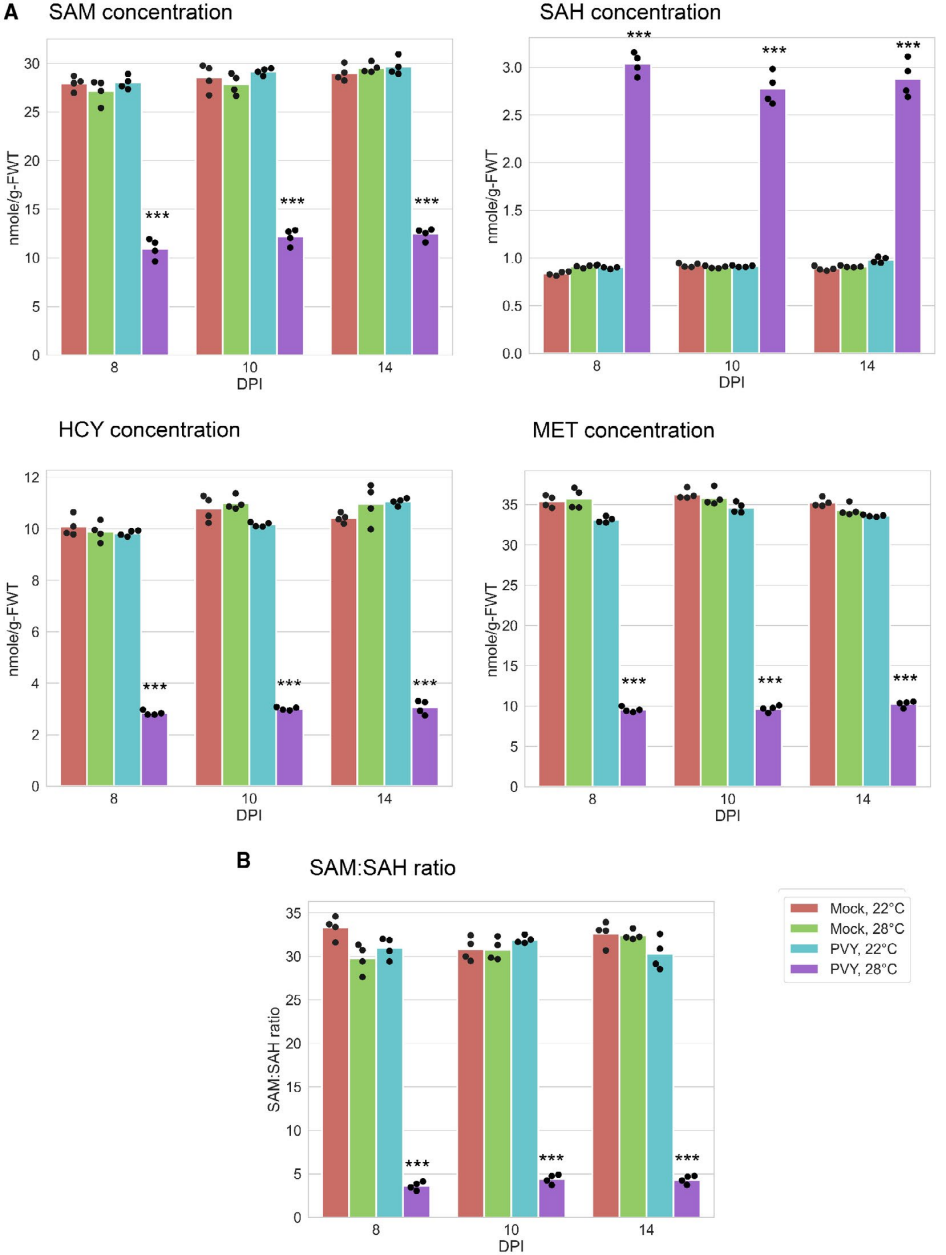
Content of methionine cycle (MTC) metabolites in systemically infected leaves of potato plants at 22 or 28 °C at 8, 10, and 14 days postinoculation (dpi) as shown. (a) Content of S‐adenosylmethionine (SAM), S‐adenosylhomocysteine (SAH), and homocysteine (HCY) was measured by ELISA. Content of methionine (MET) was measured using methionine assay kit (fluorometric). (b) The ratios of SAM to SAH. Analysis of variance and Tukey's HSD post hoc tests were performed for data. ***p < .001

Because SAM is the major methyl group donor in transmethylation reactions of various substances including proteins, DNAs, RNAs, and small siRNAs and miRNAs, and is also a key component for synthesis of the phytohormone ethylene (Mäkinen and De, 2019), we first examined the effect of PVY infection at elevated and normal temperatures on SAM accumulation. Our results indicated that PVY at 28 °C had significantly reduced SAM content in comparison to plants infected at 22 °C and uninfected controls; interestingly, the latter two treatments did not show any apparent differences between them (Figure 4a). At the same time, the content of SAH, which is a strong inhibitor of SAM‐dependent methyltransferases, was significantly increased in PVY‐infected plants at 28 °C, which is consistent with reduced expression of *SAHH*, which converts SAH into HCY (Figure 4a). As a result, the SAM:SAH ratio (referred to as the methylation index; Moffatt and Weretilnyk, 2001) sharply decreased, presumably affecting methylation processes (Figure 4b).

In PVY‐infected plants at the higher temperature, the reduced transcriptional levels of *SAHH*, *MS* and genes controlling the folate cycle could inhibit the synthesis of MET, which should concomitantly decrease the content of HCY. These predictions were confirmed by measuring levels of HCY and MET. In contrast, no essential differences in production of SAM, SAH, HCY, and MET were found between uninfected plants at both 22 and 28 °C as well as in plants infected with PVY at 22 °C.

These data present a clear and coherent picture showing that the reduction of SAM accumulation and the SAM:SAH ratio during PVY infection at elevated temperature is caused by aberrations in the expression of the key MTC‐related enzymes, which leads to coordinated alterations in the synthesis of the MTC metabolites (Figure 4).

### 2.5 Methionine subverts high susceptibility to PVY at elevated temperature in potato plants

**FIGURE 5:**
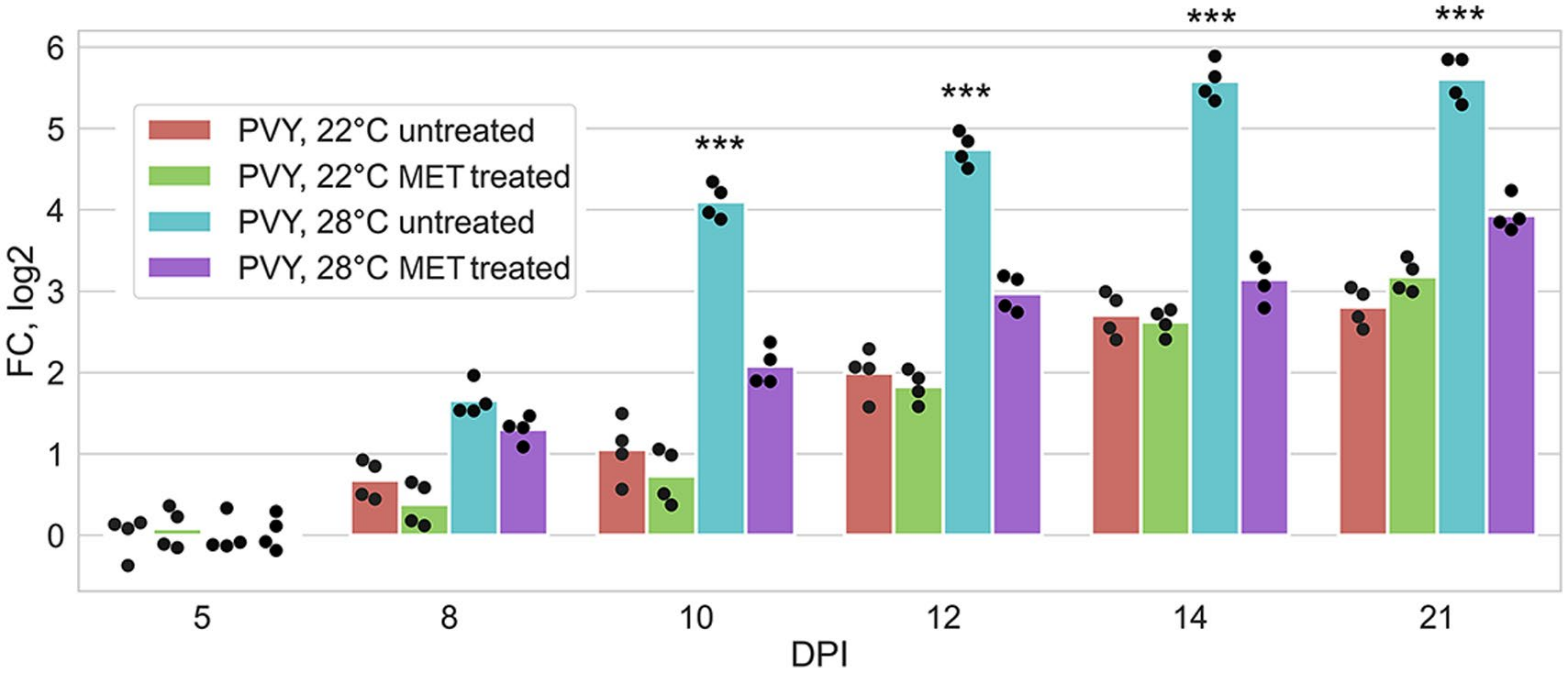
Effect of treatment with l‐methionine (MET) on accumulation of PVY RNA (measured using quantitative reverse transcription PCR [RT‐qPCR]) in systemically infected leaves of mock‐ and PVY‐inoculated potato plants at 22 or 28 °C at 5–21 days postinoculation (dpi) as shown. Analysis of variance and Tukey's HSD post hoc tests were performed for RT‐qPCR data. ***p < .001

To further investigate whether changes in the content of SAM and other MTC metabolites could confer the temperature‐sensitive susceptibility of potato plants to PVY, we employed exogenous treatment of plants maintained at 28 °C with MET, an immediate precursor of SAM. A solution of 1.5 mM MET or water was sprayed every second day onto the leaves. Following treatment with MET, accumulation of PVY RNA in systemically infected leaves of these plants dramatically decreased relative to water controls (Figure 5). In contrast, MET treatment did not significantly affect PVY RNA accumulation at 22 °C (Figure 5).

**FIGURE 6:**
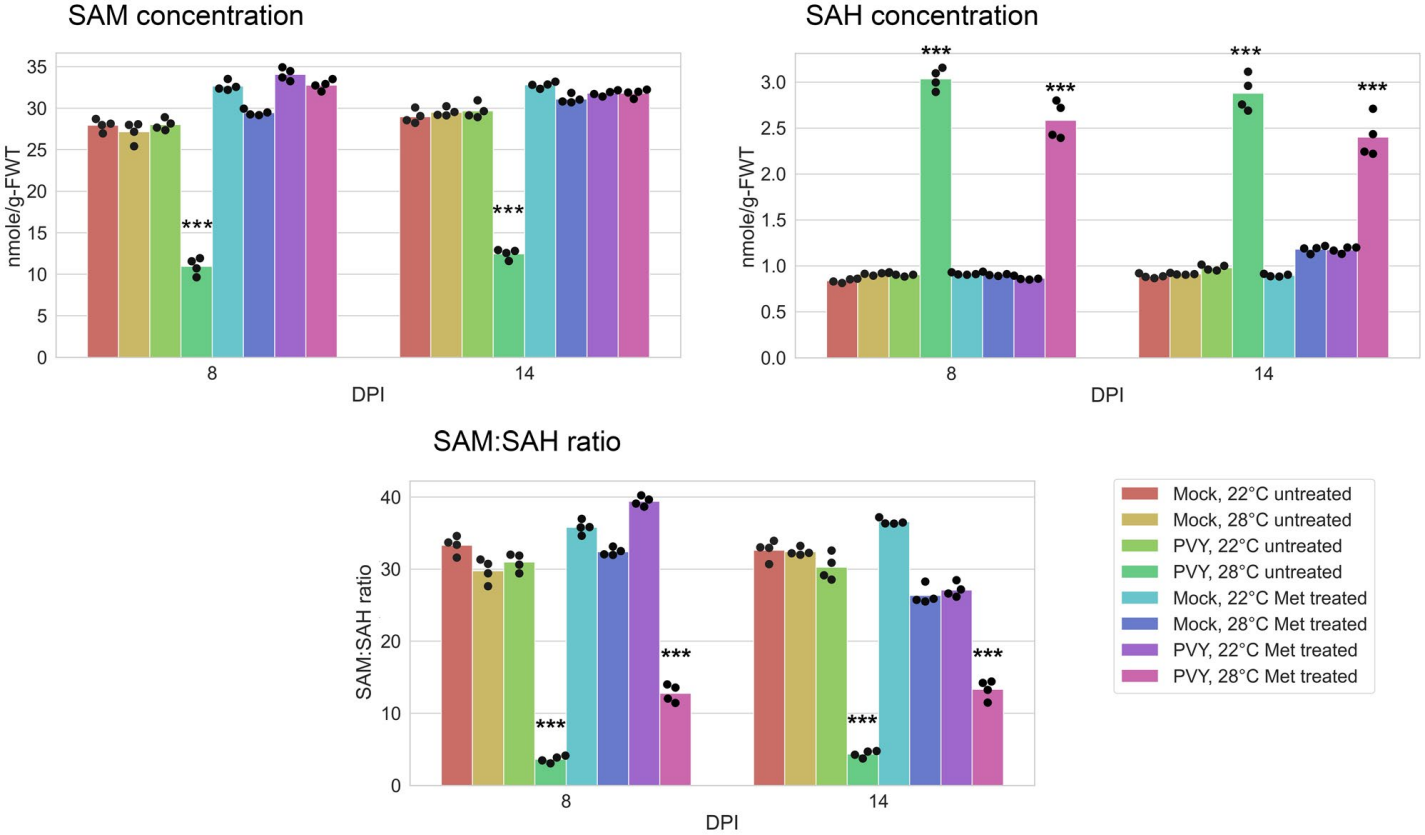
Effect of treatment with l‐methionine (MET) on accumulation of S‐adenosylmethionine (SAM), S‐adenosylhomocysteine (SAH), and SAM to SAH ratios in systemically infected leaves of potato plants at 22 or 28 °C at 8, and 14 days postinoculation (dpi) (measured by ELISA) as shown. Analysis of variance and Tukey's HSD post hoc tests were performed for data. ***p < .001

In accordance with these data, we found that MET treatment of PVY‐infected plants maintained at 28 °C had increased accumulation of SAM relative to the levels observed in mock‐inoculated (at both 22 °C or 28 °C) or PVY‐inoculated plants at 22 °C, whereas SAH concentration did not change under these conditions (Figure 6). As a result, the SAM:SAH ratio, which is indicative of methylation rates, was significantly elevated in MET‐treated PVY‐infected plants at the higher temperature relative to the untreated controls (Figure 6). This suggests that externally supplied MET could compensate for the lower expression of the key MTC enzymes and return the methylation capacity of the MTC back to normal. Interestingly, MET treatment of plants mock‐inoculated (at either 22 °C or 28 °C) or inoculated with PVY at 22 °C did not significantly affect the accumulation of MTC metabolites, implying that under these conditions the MTC operates at the optimal level and cannot be further intensified.

Collectively, these data confirm the role of the MTC in modulating PVY susceptibility in potato plants at the higher temperature.

## 3 DISCUSSION

The warming global climate is leading to rising temperatures, which may modulate plant–virus interactions, potentially further incurring significant decreases in crop yield and quality (Pandey et al., 2017). It is therefore crucial to study the effect of elevated temperatures on plant responses to virus infections.

We have recently shown that susceptibility to PVY dramatically increases in systemically infected leaves of potato at higher temperatures (Makarova et al., 2018). Similar effects have also been observed for many other viruses (e.g., Anfoka et al., 2016; Prasch and Sonnewald, 2013).

Several reports have demonstrated successful approaches using proteomics to elucidate virus resistance mechanisms in plants (Obrępalska‐Stęplowska et al., 2015; Stare et al., 2017). There is a growing body of information showing that changes in proteome and posttranslational protein modifications are directly involved in the plant immune response. However, until now information on proteomic changes in virus‐infected plants exposed to higher temperatures was scant. To compare proteomic changes in PVY‐infected plants maintained at higher and normal temperatures, we used iTRAQ‐based proteomic technology, which enables relative and absolute quantitation of proteins by labelling samples with isotope encoded reporter ions (Muth et al., 2010).

At normal temperature, we observed a rather weak proteomic response of potato plants to PVY infection. At 8 dpi, plants responded by up‐regulation of SOD, fibrillin 8 (both are known to be induced by abiotic stress conditions; Yang et al., 2006; Younus, 2018), and histone H2A. Among down‐regulated DEPs were ribosomal proteins and eukaryotic initiation factor 4A‐2 required for translation processes, suggesting possible suppression of protein synthesis. Other proteins showing decreases in expression include glutamine cyclotransferase and monodehydroascorbate reductase, which are associated with glutathione metabolism (Hasanuzzaman et al., 2019). Glutathione is known to be an antioxidant and may play an important role in plant defence signalling against biotic and abiotic stresses (Hasanuzzaman et al., 2019). At 14 dpi, the overall degree of proteomic modulation in response to PVY infection was at similar magnitude to that observed at 8 dpi, but there were some differences in the levels of some proteins. For example, at 14 dpi, SOD was down‐regulated and ribosomal proteins were up‐regulated in comparison to the levels observed at 8 dpi, but the biological relevance for their up‐regulation remains unclear.

At elevated temperature, the proteome changes were much more pronounced. Proteins in the up‐regulated group were mainly related to stress responses, such as calreticulin, salt tolerance protein 4, acidic endochitinase, patatin 3, glutathione S‐transferase, glyoxysomal fatty acid β‐oxidation multifunctional protein, and small heat‐shock proteins (Table S3). Given high rates of PVY accumulation under these conditions, these proteome changes may be attributed to general host responses to plant stress/disease induced by PVY infection at the higher temperature, but these responses are not sufficient to restrict virus invasion. Down‐regulation of ribosomal proteins under these conditions may reflect a relatively low level of viral translation at this stage of the infection. Identification of down‐regulated DEPs associated with MTC seemed to us the most striking finding of this work and therefore we investigated its role in the plant response to PVY in more detail (see below).

As stated above, the later stage of PVY infection (14 dpi) at the higher temperature results in up‐regulation of different stress‐ and pathogen‐responsive (defensive) proteins, which do not compromise virus accumulation, given the high virus titres observed. This confirms the suggestion that the increase in the expression of these proteins mirrors a general response to stress induced by PVY at 28 °C rather than a specific productive defence reaction. The proteomic changes occurring at this late stage of highly productive infection may reflect large damaging effects on the core biological processes in plants, including photosynthesis and carbon metabolism. Some of these effects may be collateral damage caused by general stress and defence responses during infection, particularly at elevated temperatures.

**FIGURE 7:**
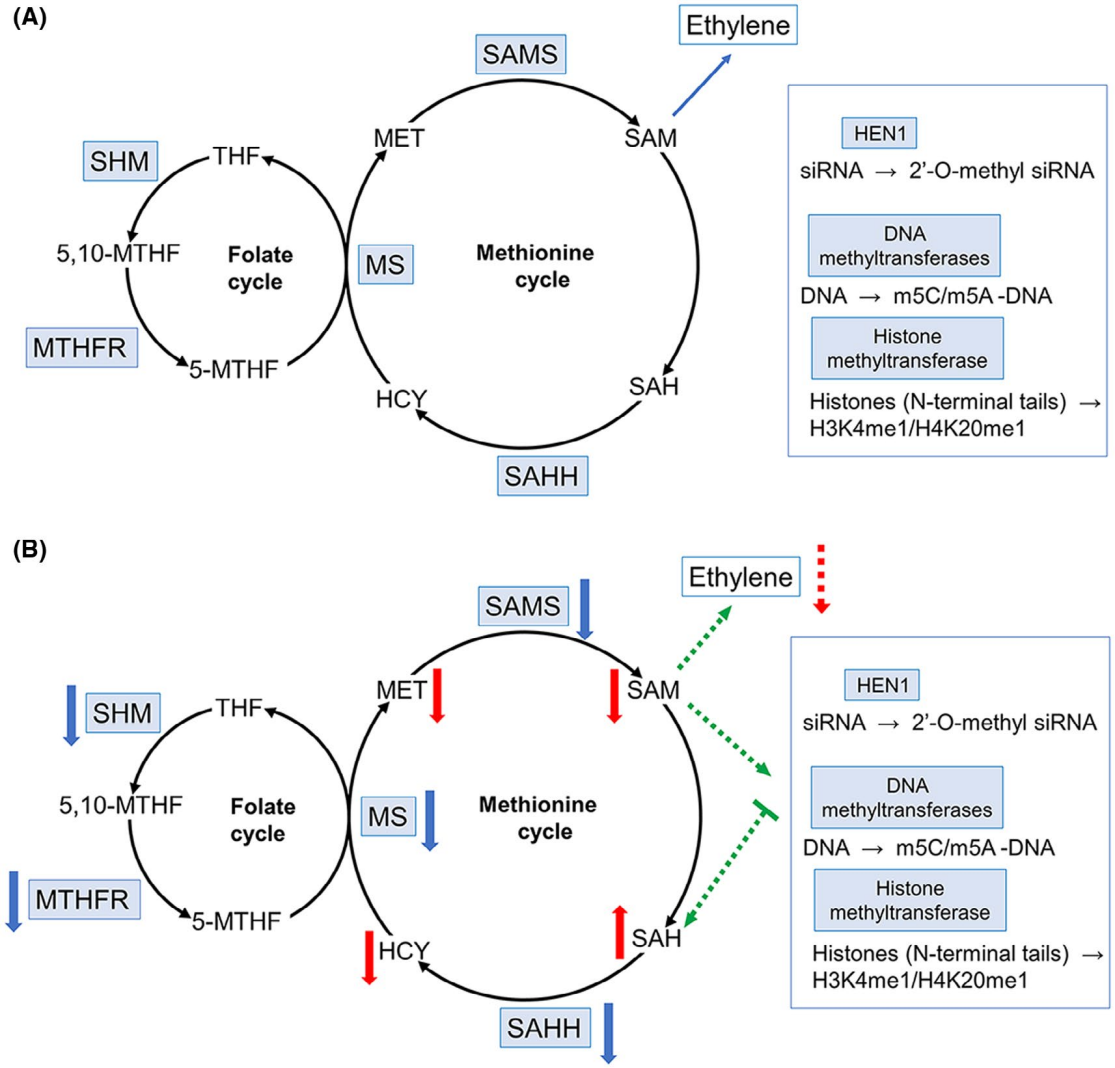
A tentative model showing possible mechanisms underlying the temperature‐sensitive susceptibility of potato plants to PVY under elevated temperature. (a) Schematic representation of the methionine cycle (MTC) in healthy cells. SAMS converts methionine (MET) to S‐adenosylmethionine (SAM), which serves as a methyl donor for transmethylation reactions of various substances including proteins, DNAs, and small interfering (si) RNAs and is also a key component for the synthesis of ethylene. The methylation reaction by‐product S‐adenosylhomocysteine (SAH) is consequently hydrolysed by SAHH to adenosine and homocysteine (HCY). Then HCY is converted back to MET by MS. (b) PVY infection at higher temperature causes down‐regulation of key MTC (MS, SAMS, SAHH) and MTC‐related folate cycle (SHM, MTHFR) enzymes, leading to the decrease of SAM and increase of SAH. This would suppress SAM‐dependent methylation reactions, including HEN1‐directed methylation of siRNAs reducing stability of antiviral siRNAs. Inhibition of DNA and protein methyltransferases may also contribute to the susceptibility response to PVY. Finally, a decrease in ethylene production may be another factor conferring increased susceptibility to PVY at higher temperatures. Decreasing and increasing levels of MTC and MTC‐related enzymes and metabolites are indicated by arrows

How these virus and temperature‐induced variations in the proteome are realized physiologically is complex to appraise from a mechanistic perspective, and in consequence we selected one of the most affected pathways (MTC; Figure 7a) for experimental validation.

The orchestrated down‐regulation of levels of major enzymes involved in the MTC (MS, SAMS, SAHH) and MTC‐related folate cycle (SHM and MTHFR) in potato leaves systemically infected with PVY observed at the early stage (8 dpi) of infection at 28 °C is perhaps one of the most intriguing findings of this study. It is important that the proteomic data indicating differential expression of these proteins are consistent with the expression rates of the corresponding genes under the same conditions. As would be expected, a decrease in the expression levels of SAHH, MS, SHM, MTHFR, and SAMS led to reduction of HCY, 5′‐MTHF, and MET as sequential precursors of SAM (Figure 7b), and consequently led to a decrease in the levels of SAM itself as a methyl donor of methyltransferases. At the same time down‐regulation of SAHH caused increased levels of SAH, which is a substrate for SAHH (Figure 7b). Concomitant decreases in the SAM:SAH ratio, which is commonly considered an indicator of cellular methylation potential, could be expected to inhibit transmethylation (Mäkinen and De, 2019; Moffatt and Weretilnyk, 2001). The correlation between the high susceptibility of potato plants to PVY at the higher temperature and down‐regulation of the expression of the MTC‐related enzymes at both protein and RNA levels combined with relevant changes in accumulation rates of MTC metabolites provides an indication of the functional relationship between the temperature‐sensitive susceptibility of the plants to PVY and operation of the MTC. This is further supported by the coincidental ability of exogenous MET to reinstate operation of the MTC (restoring the normal levels of its metabolites) and reduce susceptibility to PVY at elevated temperatures in potato plants. In this connection, it is worth noting that exogenous MET treatment may constitute a powerful approach to the modulation of the plant defence response against biotic and abiotic stresses.

With regards to virus–plant interactions, SAM‐mediated transmethylation is closely linked to RNAi pathways in which siRNAs (as major components of RNAi pathways) are stabilized by the MTC‐associated methyl transferase Hua enhancer 1 (HEN1), which 2′‐O‐methylates the 3′ terminal nucleotides of siRNAs using SAM as a donor (Li et al., 2005). One of the possible mechanisms by which higher temperatures increase plant (potato) susceptibility to PVY may be depletion of siRNA methylation by HEN1 due to the reduced SAM:SAH ratio. Moreover, it is well known that antiviral RNAi‐based defence against potyviruses, including PVY and its close relative potato virus A (PVA), is suppressed by the potyviral helper component‐proteinase (HC‐Pro) (Ivanov et al., 2016). Interestingly, HC‐Pro has also been demonstrated to physically interact with SAMS and SAHH (Canizares et al., 2013; Ivanov et al., 2016), possibly further reducing their activities (in addition to down‐regulation). Thus, down‐regulation of SAHH and SAMS and interaction of HC‐Pro with both these enzymes could additively increase the SAM:SAH ratio, leading to decreased methylation and further destabilization of PVY‐specific siRNA. This may explain the increased susceptibility of potato plants to PVY at the higher temperature.

Another possibility would be a change in plant DNA methylation (Figure 7b), which is one of the epigenetic factors that regulates gene expression. DNA methylation is controlled by several plant SAM‐dependent DNA methyltransferases and therefore may be affected by perturbations in the MTC (Correa et al., 2020; Kuznicki et al., 2019). To fulfil its functions in plant development and responses to biotic and abiotic stress conditions, DNA methylation in plants normally achieves a balance between stable and flexible methylation status tuned with the transcriptional repressive or active state of the corresponding genes. This process is highly complex, given that the epigenetic activity of different methyltransferases may be differentially sensitive to changes in the methylation index. The role of pathogens, and especially RNA viruses in DNA methylation responses, remains largely uncharacterized (Correa et al., 2020; Kuznicki et al., 2019). Therefore, our future efforts will be focused on the elucidation of the regulatory role of specific small RNA and DNA methylation activities in the mechanisms of plant–virus resistance/susceptibility. Protein/histone methylation may also be involved in the response of potato plants to PVY at higher temperatures (Figure 7b).

An additional factor that may contribute to the increased susceptibility of potato plants to PVY at higher temperatures is a potential change in ethylene production, which is also associated with the MTC. Ethylene, like other phytohormones, plays an important role in plant responses to various pathogens, including viruses. Moreover, it is widely accepted that ethylene may play a significant role in triggering different types of acquired resistance (Alazem and Lin, 2015; Van Loon et al., 2006). Thus, greater susceptibility of potato plants to PVY at the higher temperature may be determined, for example, by the lack of the MTC metabolites needed for ethylene production.

Our data show that neither PVY infection at normal temperature (on its own) nor elevated temperature alone induce alterations in the expression of MTC‐related genes. Only combined stress caused by PVY and the higher temperature show a significantly different expression pattern for these genes (down‐regulation) and consequent relevant changes in the levels of MTC metabolites. These data are in good agreement with many other reports showing that a quite distinct gene expression programme may be established in response to combined stress as a result of integration of individual stress‐responsive pathways (Makarova et al., 2018; Prasch and Sonnewald, 2013; Rizhsky et al., 2004). Thus, we suggest that responses to combined heat stress and PVY infection in potato cv. Chicago are integrated and reprogrammed in a way to specifically affect the MTC in a manner which increases plant sensitivity to PVY.

We have also previously shown that SA, another essential component of regulatory defence signalling pathways, affects susceptibility to PVY in cv. Chicago plants maintained at the higher temperature (Makarova et al., 2018). The molecular mechanisms underlying the functional links between MTC and SA‐dependent responses remain unknown. A possibility is that both MTC and SA are interconnected with regulation of RNAi (Alamillo et al., 2006; Canizares et al., 2013; Ding et al., 2001; González et al., 2012; Ivanov et al., 2016; Jovel et al., 2011; Lee et al., 2016; Li et al., 2005; Mäkinen and De, 2019). Some other findings point to intimate interplay between SA‐mediated defence and signalling pathways directed by ethylene (Li et al., 2019) that is synthesized under control of MTC. It also cannot be ruled out that MTC and SA operate independently each of other in conferring virus resistance/susceptibility.

Thus, it is unlikely that controlling resistance or susceptibility of plants to viruses relies on a single regulatory mechanism. The interplay between various mechanisms may be integrated into specific consolidated networks perhaps allowing the plant to fine‐tune defence responses under certain environmental conditions.

## 4 EXPERIMENTAL PROCEDURES

Additional details can be found in the Supporting Information Methods S1.

### 4.1 Virus, plants, and growth conditions

Ordinary PVY strain O (PVY^O^; Gibson et al., 1990), hereinafter referred to as PVY, was maintained in *Nicotiana tabacum*. Potato plants (*S. tuberosum* 'Chicago') were inoculated with PVY and maintained for the duration of the experiments in controlled growth chambers (Pol‐Eko‐Aparatura, Poland) with a 16/8 hr day/night photoperiod at a relative humidity of 60% with a light fluence of 250 µmol⋅m^−2^⋅s^−1^. At 2 dpi, half of the plants were transferred to 28 °C, while the other half remained at 22 °C. Tissue samples from systemically infected leaves of three mock‐ and virus‐inoculated plants (two leaves per plant) were collected and pooled together at different time points for each of four independent experiments and used for various types of analyses as documented below.

### 4.2 Protein extraction and trypsin digestion

Proteins were extracted using the phenol extraction method (Faurobert et al., 2007), quantified by Bradford protein assays (Bio‐Rad), and reduced in 5 mM dithiothreitol for 30 min at 50 °C and then alkylated by incubating with iodoacetamide. Obtained proteins were digested by incubating with trypsin (Promega). Peptide labelling was conducted according to manufacturer's recommendations for the 8‐plex iTRAQ kit (ABsciex Inc.). The samples collected at 8 dpi were labelled as follows: iTRAQ reagents 118, 119, 121 and 115, 116, 117 were used to label mock‐inoculated samples (Mock22); iTRAQ reagents 113, 114, 117 were used to label peptides from virus‐infected plants grown at 22 °C (PVY22) and 118, 119, 121 at 28 °C (PVY28). Mock‐inoculated samples collected at 14 dpi were labelled by iTRAQ reagents 113, 114, 115; the corresponding PVY22 and PVY28 samples were labelled by 116, 117, and 121 isobaric tags. The labelled peptides were combined as follows: Mock22 (118, 119, 121) with PVY22 (113, 114, 117) and Mock22 (115, 116, 117) with PVY28 (118, 119, 121) for 8 dpi samples; Mock22 (113, 114, 115) with PVY22 (116, 117, 121) or PVY28 (116, 117, 121) for 14 dpi samples.

### 4.3 LC‐MS/MS analysis and protein identification and quantification

Peptides were separated on Acclaim PepMap 100 C18 (75 μm × 50 cm; Thermo Fisher Scientific). Reverse‐phase chromatography was performed with an Ultimate 3000 Nano LC System (Thermo Fisher Scientific), which was coupled to the Q Exactive HF benchtop Orbitrap mass spectrometer (Thermo Fisher Scientific) by a nanoelectrospray source (Thermo Fisher Scientific). Peptides were analysed, identified, and quantified as described in Methods S1. Taking into account that iTRAQ quantification usually underestimates the amount of real fold changes between two samples (Ow et al., 2009), differential protein screening (determined by the ratio in the PVY‐infected samples and untreated control [mock‐inoculated] plants at 22 °C) was performed using a fold change ratio >1.20 (for up‐regulated DEPs) or <0.83 (for down‐regulated DEPs) (*p *< .05, one‐way analysis of variance).

### 4.4 Bioinformatic analysis

Protein–protein interaction networks were constructed using STRING v. 10 (www.string‐db.org; Szklarczyk et al., 2015). The strength of protein interactions was set at 0.4, the default option. Cytoscape software (Sannon et al., 2003) was applied to visualize the protein interaction networks. The functions and pathway enrichment of candidate DEGs were analysed using g:Profiler (Raudvere et al., 2019).

### 4.5 RNA extraction and RT‐qPCR

Species‐specific prefixes (St) and figure legends to define mRNAs corresponding to the *S. tuberosum* genes are used in this section: *StMS*, *StSAMS*, *StSAHH*, *StSHM*, *StMTHFR*, *StEF‐1α*, and *StCox*. However, for simplicity, in the main body of the manuscript the “St” nomenclature is not used for genes, proteins, or mRNAs. Total RNA was isolated as described previously (Makarova et al., 2018). Aliquots of DNase‐treated RNA were reverse transcribed into cDNA using the SuperScript First‐Strand Synthesis System for RT‐PCR (Invitrogen), in conjunction with either an oligo‐dT primer (for host plant‐specific mRNAs) or a PVY‐specific primer (see Table S1). The primer pairs for SYBR Green‐based real‐time PCR analysis of PVY RNA and host mRNAs were designed using Plant Genomics Resource Phytozome 12 (https://phytozome.jgi.doe.gov/pz/portal.html) and PRIMER EXPRESS software, and are listed in Table S1. The *C*_t_ values for PVY RNA and each mRNA of interest were normalized using two internal reference genes encoding cytochrome c oxidase subunit 1 (StCOX; Baebler et al., 2011) and StEF‐1α (Nicot et al., 2005); primers are listed in Table S1. The average of the *C*_t_ values of the two reference genes was used to analyse PVY and host mRNA levels. More detailed information on the RT‐qPCR procedure is provided in Methods S1.

### 4.6 Analysis of MTC‐related metabolites

To detect and quantify the contents of MET, SAM, SAH, and HCY, approximately 50 mg of fresh tissue samples from infected leaves of three mock‐ and virus‐inoculated plants were collected, pooled together, and ground in phosphate‐buffered saline (0.01 M, pH 7.2). After centrifuging at 12,000 × *g* for 15 min, the extracts were used for analyses. The content of SAM, SAH, and HCY was measured by ELISA using an S‐adenosylmethionine (SAM) and S‐adenosylhomocysteine (SAH) ELISA Combo Kit and a Homocysteine (HCY) ELISA Kit (Cell Biolabs, Inc.), respectively, according to the manufacturer's instructions. MET was quantified using a methionine (fluorometric) assay kit (Abcam) according the manufacturer's instructions.

### 4.7 Statistics

Statistical analysis was performed across four biological repeats. Statistical analyses and bar plots were made in Python v. 3.7.5 (G. van Rossum, Python tutorial, Technical Report CS‐R9526, Centrum voor Wiskunde en Informatica (CWI, Amsterdam, May 1995). For two‐ or more‐way analysis of variance (ANOVA), Tukey's honestly significant difference (HSD) tests based on multiple comparisons of means were applied to determine which pairwise comparisons were statistically significant. Differences were considered to be significant at *p* ˂ .05.

### 4.8 Foliar (exogenous) application of MET

A solution of 1.5 mM l‐methionine (Merck) or water (as control) was sprayed every second day onto the leaves of mock‐ and PVY‐infected plants maintained at 22 or 28 °C. PVY RNA accumulation and content of MTC‐related metabolites in these plants were quantified as described above.

## CONFLICT OF INTEREST

The authors declare no conflict of interest.

## Supporting information


**TABLE S1** Primers used for quantitative RT‐PCR

**TABLE S2** The list of identified proteins

**TABLE S3** The list of differentially regulated proteins

**TABLE S4** The results of functional enrichment analysis by g:Profiler

**METHODS S1** Supplementary methods

## Data Availability

The mass spectrometry proteomics data have been deposited to the ProteomeXchange Consortium at http://www.proteomexchange.org/ via the PRIDE [1] partner repository with the data set identifier PXD020495. The other data that support the findings of this study are available from the corresponding author upon reasonable request.
