# Supplementary material for: Role of the methionine cycle in the temperature‐sensitive responses of potato plants to potato virus Y
Source: Mol Plant Pathol. 2020 Nov 4;22(1):77–91. doi: 10.1111/mpp.13009 (PMC7749756; doi:10.1111/mpp.13009)
Supplement: Supplementary file 1 — TABLE S1 Primers used for quantitative RT‐PCR [file MPP-22-77-s001.docx]

**Table S1**. Primers used for quantitative RT PCR

| **Primer** | **5’-3’ sequence** | **Reference** | **Primer concentration (nM)** | **E (%)** |
| --- | --- | --- | --- | --- |
| PVY^O^-F  PVY^O^-R | TATGATGGATTTGGCGACCACTTGT  TAAACTAGGCAGCTCTGCATCATG | Makarova et al., 2018 | 400 | 95.7 |
| StMS-F  StMS-R | GTTAGTGAATACAAGGAGGCT  GCTGCTTTTGATAACAAGAGG | PGSC0003DMP400015309* | 400 | 98.5 |
| StSAMS-F  StSAMSR | ATCACGACCAAGGCTATTGT  CAGGACTTTGCTGCTCAATG | PGSC0003DMP400031888* | 400 | 94.5 |
| StSAHH-F  StSAHH-R | AACTTTGCTTTTCCCTGCT  CGGCAATAAGGGCAACC | PGSC0003DMP400008076* | 350 | 94.5 |
| StSHM-F  StSHM-R | AGGTTGCTGATAAATGTGGG  CTCTTGTGTGTGGTGGTAG | PGSC0003DMP400027608* | 350 | 93. 4 |
| StMTHFR-F  StMTHFR-R | GTGGATTTGCTTGTGCTCT  TGTGCCTCTGGATAACCTG | PGSC0003DMP400028247* | 300 | 97.5 |
| StCOX-F  StCOX-R | GGTCGGACATACCTGAAAC  CCAAAAGTATGAAAAGCTGGAG | Baebeler et al., 2011 | 350 | 97.3 |
| StEF-1α-F  StEF-1α-R | CTTGACGCTCTTGACCAGATT  GAAGACGGAGGGGTTTGTCT | Nicot et al., 2005 | 350 | 98.7 |

Full references are provided in the main text. Primer concentrations giving the lowest threshold cycle (C_t_) value were utilized in RT-PCR and are listed in the Table. E, efficiency of PCR amplification as calculated by CFX Manager Software. *Gene sequences were retrieved from Plant Genomics Resource Phytozome 12 (<https://phytozome.jgi.doe.gov/pz/portal.html>).
