# Supplementary material for: Role of the methionine cycle in the temperature‐sensitive responses of potato plants to potato virus Y
Source: Mol Plant Pathol. 2020 Nov 4;22(1):77–91. doi: 10.1111/mpp.13009 (PMC7749756; doi:10.1111/mpp.13009)
Supplement: Supplementary file 5 — METHODS S1 Supplementary methods [file MPP-22-77-s005.docx]

**Supplementary Methods**

**Virus, plants and growth conditions**

Ordinary PVY strain O (PVY^O^; Gibson *et al*., 1990) hereinafter referred to as PVY, was maintained and propagated in *Nicotiana tabacum.* Potato plants (*S. tuberosum* L.; cultivar Chicago) were grown and maintained by micro-propagation in stem node tissue culture. Two weeks after node segmentation, they were transferred to soil and after one month of growth were used for heat treatment and PVY infection experiments. Plants were maintained for the duration of the experiments in controlled growth chambers (Pol-Eko-Aparatura, Poland) with a 16/8 hour day/night photoperiod at a relative humidity of 60% with a light fluence of 250 µmol m^-2^ s^-1^. To prepare PVY inoculum, PVY-infected *N. tabacum* plant leaf material was ground in liquid nitrogen to a fine powder using a mortar and pestle, after which cold 125 mM potassium phosphate (K-P) buffer pH 7.5 was added at a ratio of 1:3 (w/v). The extract was centrifuged at 13000 g for 10 minutes at 4°С and the supernatant was used for inoculation. For inoculation, two leaves of each plant were dusted with celite and rubbed with either 50 µL of virus inoculum or K-P buffer pH 7.5 (for mock inoculated controls). Inoculated leaves were then twice washed with water. Two days post inoculation (dpi) half of the plants were transferred to 28°C (elevated temperature); while the other half remained at 22°C. Tissue samples from systemically infected leaves of three mock- and virus-inoculated plants (two leaves per plant) were collected and pulled together at different time points [3, 5, 8, 10, 12, 14, 16 and 21 dpi) and used for various types of analyses as documented below.

**Protein extraction and trypsin digestion**

Proteins were extracted using the phenol extraction method (Faurobert *et al.,* 2007). Plant tissue was homogenized in ice-cold extraction buffer (500 m Tris–HCl, pH 8.0, 50 mM EDTA, 700 mM sucrose, 100 mM КCl, 1 mM phenylmethylsulfonyl fluoride, 1 mM DTT), followed by 10 min incubation on ice. An equal volume of ice-cold Tris–HCl (pH 8.0)-saturated phenol was added, and the mixture was vortexed and incubated for 10 min with shaking. After centrifugation (10 min, 5500 × g, 4°C), the phenol phase was collected and re-extracted with extraction buffer. Proteins were precipitated from the final phenol phase with three volumes of ice-cold 0.1 M ammonium acetate in methanol overnight at −20°C. The pellets were rinsed with ice-cold 0.1 M ammonium acetate in methanol three times and with ice-cold acetone once and then dried. The resulting pellet was dissolved in 8 M urea, 2 M thiourea and 10 mM Tris, and the proteins were quantified via Bradford protein assays (Bio-Rad, Hercules, CA USA). One hundred micrograms of protein were reduced in 5 mM DTT for 30 min at 50°C and then alkylated by incubating with 10 mM iodoacetamide for 20 min at room temperature. Obtained proteins were five times diluted with 40 mM ammonium bicarbonate and digested by incubating with 1 μg sequence-grade modified trypsin (Promega, Madison, WI, USA) at 37 ˚C overnight. The reaction was stopped by adding trifluoroacetic acid to a concentration of 1%. Twenty micrograms of each sample were desalted using Empore octadecyl C18 extraction disks (Supelco by Sigma-Aldrich, St. Louis, MO, USA) and then dried in a vacuum concentrator. iTRAQ labelling (Applied Biosystems, Foster City, CA, USA) was conducted according to manufacturer’s recommendations. Proteins were labeled with the iTRAQ tags as follows: Mock 22 – 116, 117, 118 isobaric tags, PVY 22  – 113, 114, 117 and PVY 28 – 113, 114, 115 isobaric tags for 8 dpi; Mock 22 – 113, 114, 115 isobaric tags, PVY 22 and PVY 28 – 116, 117, 121 isobaric tags for 14 dpi. The labeled peptides were mixed, cleaned using SCX membranes (Thermo Fisher Scientific, MA, USA) and then vacuum dried.

**LC-MS/MS analysis**

Peptides were separated on Acclaim PepMap 100 С18 (75 μm x 50-cm) (Thermo Fisher Scientific MA, USA). Reverse-phase chromatography was performed with an Ultimate 3000 Nano LC System (Thermo Fisher Scientific, MA, USA), which was coupled to the Q Exactive HF benchtop Orbitrap mass spectrometer (Thermo Fisher Scientific, MA, USA) via a nanoelectrospray source (Thermo Fisher Scientific, MA, USA). Peptides in 5 μl of loading buffer (3% (v/v) acetonitrile, 0.1% (v/v) TFA in Milli-Q deionized water) were loaded on a trapping column PepMap 100 C18 (0.1 x 20 mm) (Thermo Fisher Scientific MA, USA) at a flow rate of 5 μl/min for 6 min. NanoLC pump mobile phases were: A - 2% (v/v) acetonitrile, 0.1% (v/v) formic acid in Milli-Q deionized water; B - 80% (v/v) acetonitrile, 0.1% (v/v) formic acid, 19.9% (v/v) milli-Q deionized water. Peptides were eluted from trapping column with a linear gradient: 5–28% B for 90 min; 28-45% B for 20 min, and 45-100% B for 7 min at a flow rate of 350 nl/min. After each gradient, the column was washed with 100% buffer B for 5 min and re-equilibrated with buffer A for 10 min. Peptides were analyzed on a mass spectrometer, with one full scan (375–1400 m/z, R = 120,000 at 200 m/z) at a target of 3e6 ions and max ion fill time 50 ms, followed by up to 15 data-dependent MS/MS scans with higher-energy collisional dissociation (HCD) (target 10^5^ ions, max ion fill time 100 ms, isolation window 1.2 m/z, normalized collision energy (NCE) 32%), detected in the Orbitrap (R = 30,000 at fixed first mass 100 m/z). Other settings: charge exclusion - unassigned, 1, >6; peptide match – preferred; exclude isotopes – on; dynamic exclusion - 60 s was enabled.

**Protein identification and quantification**

Tandem mass spectra were analyzed by PEAKS Studio version 8.0 software (Bioinfor Inc., CA, USA). The custom database was built from Phytozome database *Solanum tuberosum* combined with chloroplast and mitochondrial proteins (39,809 records). The database search was performed with the following parameters: a fragmentation mass tolerance of 0.05 Da; parent ion tolerance of 10 ppm; fixed modification – carbamidomethylation; variable modifications - oxidation (M), deamidation (NQ), and acetylation (Protein N-term). The results were filtered by a 1% false discovery rate (FDR). PEAKS Q was used for iTRAQ quantification. Normalization was performed by averaging the abundance of all peptides. Median values were used for averaging. Differentially expressed proteins were filtered if their fold change was greater than 1.2 and significance threshold 15. contained two unique peptides with a statistical P-value (ANOVA test with Benjamini and Hochberg FDR correction) below 0.05, variance homogeneity test (P-value > 0.05), and normal distribution test (P-value > 0.05).

**RNA Extraction and Real Time Quantitative RT-PCR (qRT-PCR)**

Species-specific prefixes (St) are used in this section and figure legends to define mRNAs corresponding to the *S. tuberosum* genes: *StMS, StSAMS, StSAHH, StSHM, St MTHFR, StEF-1α* and *StCox.* However, for simplicity, in the main body of the manuscript this “St” nomenclature is not used for *S. tuberosum* genes, proteins or mRNAs. Leaf tissue was ground into a fine powder under liquid nitrogen using a mortar and pestle, and RNA was extracted from the grindate using the MagMAX™ Plant RNA Isolation Kit (ThermoFisher Scientific, MA, USA) according to manufacturer recommendations. The purity of the RNA samples was determined by absorbance readings at 260/280 nm, and the RNA integrity was verified by electrophoresis in a 1% agarose 1xTBE gel stained with ethidium bromide. Residual DNA was removed by treating RNA with the RNase-free DNase I kit according to the manufacturer’s protocols (Invitrogen, Schwerte, Germany). Aliquots of 2 µg of DNase-treated RNA were reverse transcribed into cDNA using the SuperScriptTM First-Strand Synthesis System for RT-PCR (Invitrogen), in conjunction with either an oligo-dT primer (for host plant-specific mRNAs) or a PVY specific primer (see Table S1). The primer pairs for SYBR green-based real-time PCR analysis of PVY RNA and host mRNAs (StMS mRNA, StSAMS mRNA, StSAHH mRNA, StSHM mRNA and StMTHFR mRNA) were designed using Plant Genomics Resource Phytozome 12 (<https://phytozome.jgi.doe.gov/pz/portal.html>) and PRIMER EXPRESS software, and are listed in Table S1. Primer concentrations giving the lowest threshold cycle (C_t_) value were utilized in RT-PCR and are also shown in Table S1. Real-time RT-PCR was carried out in an ABI PRISM 7700 Sequence Detection System (Applied Biosystems, USA) on 10-fold dilutions of first-strand cDNA reaction mixes using the procedures described in the QuantiTect^TM^ SYBR^®^ Green PCR kit (Qiagen). All reactions were heated to 95^o^C for 15 min, followed by 40 cycles of 94^o^C for 15 sec, 60^o^C for 30 sec and 72^o^C for 30 sec. The C_t_ values for PVY RNA and each mRNA of interest were normalized using two internal reference genes encoding cytochrome c oxidase subunit 1 (StCOX; Baebler *et al*., 2011) and StEF-1α (Nicot *et al.,* 2005); primers are listed in Table S1. Fluorescence signals from each sample were recorded and analysed using StepOne™ Software v2.1 (Applied Biosystems). Each pooled RNA sample was analysed with three replicate reactions. The comparative Ct (ΔΔ Ct) method was employed to calculate PVY and host mRNA levels on the StepOnePlus™ Real-Time PCR System (Applied Biosystems).

**For references see Main Text.**
